# T Cell Immunity against Influenza: The Long Way from Animal Models Towards a Real-Life Universal Flu Vaccine

**DOI:** 10.3390/v13020199

**Published:** 2021-01-28

**Authors:** Anna Schmidt, Dennis Lapuente

**Affiliations:** Institute of Clinical and Molecular Virology, University Hospital Erlangen, Friedrich-Alexander University Erlangen-Nürnberg, 91054 Erlangen, Germany; Anna.Schmidt@uk-erlangen.de

**Keywords:** influenza, vaccine, influenza vaccine, T cells, tissue-resident memory T cells, TRM, universal flu vaccine

## Abstract

Current flu vaccines rely on the induction of strain-specific neutralizing antibodies, which leaves the population vulnerable to drifted seasonal or newly emerged pandemic strains. Therefore, universal flu vaccine approaches that induce broad immunity against conserved parts of influenza have top priority in research. Cross-reactive T cell responses, especially tissue-resident memory T cells in the respiratory tract, provide efficient heterologous immunity, and must therefore be a key component of universal flu vaccines. Here, we review recent findings about T cell-based flu immunity, with an emphasis on tissue-resident memory T cells in the respiratory tract of humans and different animal models. Furthermore, we provide an update on preclinical and clinical studies evaluating T cell-evoking flu vaccines, and discuss the implementation of T cell immunity in real-life vaccine policies.

## 1. Introduction

Influenza viruses are a constant threat to the world community. Globally, 290,000–650,000 seasonal influenza-associated deaths are estimated with the highest mortality rate in sub-Saharan Africa and southeast Asia [1]. The classical risk groups include very young children, the elderly, and individuals with co-morbidities [2,3,4]. In addition to seasonal epidemics, influenza A viruses (IAVs) occasionally cause pandemic outbreaks. While the “Spanish flu” from 1918 was the most devastating of these pandemics, with an estimated 50 million deaths [5], the most recent H1N1 pandemic in 2009 was only moderately pathogenic. Although not caused by a flu strain, the 2020 severe acute respiratory syndrome coronavirus type 2 (SARS-CoV-2) pandemic has revealed the dramatic impact of an emerging respiratory pathogen on healthcare, social life, and the economy in the 21st century.

Influenza viruses belong to the *Orthomyxoviridae* family, and consist of the four genera A, B, C, and D, with IAV and influenza B virus (IBV) being most relevant for human disease. IBV has a limited host range and strain diversity (Yamagata and Victoria lineages), and does not cause pandemics. In contrast, the genetic instability of IAV constantly creates new virus lineages and subtypes. The error-prone viral polymerase of IAV and IBV lacks a proofreading activity, leading to a continuous accumulation of mutations, especially in the surface proteins hemagglutinin (HA) or neuraminidase (NA) [6,7], while the internal virus proteins remain more conserved. This phenomenon called “genetic drift” allows the genetic evolution of seasonal flu strains. “Genetic shift” occurs only in IAV, and describes the exchange of one or more gene segments among different IAV strains upon superinfection, leading to novel virus subtypes. By this mechanism, novel viruses can emerge against which weak or no herd immunity exists in the human population [8]. Thus, the ongoing drift of seasonal flu strains and the occasional emergence of IAV pandemics are a constant threat to the world community. While current vaccines elicit mainly strain-specific protection, there are substantial efforts to develop a universal influenza vaccine. This review summarizes recent flu vaccine strategies and their shortcomings, the potential of cross-reactive T cell responses in flu immunity, and the remaining challenges for the clinical use of T cell-evoking influenza vaccines.

## 2. Current Influenza Vaccines

Vaccines are considered to be the most cost-effective health care intervention against flu. Currently, two types of seasonal vaccines are licensed: tri- or quadrivalent inactivated vaccines (TIV/QIVs) and live-attenuated influenza vaccines (LAIVs). Both types combine antigens from two IAV (H1N1 and H3N2) and one or two IBV strains (Yamagata and/or Victoria). HA-directed neutralizing antibodies (nAbs) are the major immune correlate induced by those vaccines, and the hemagglutination inhibition assay (HAI) is routinely used to measure this correlate of protection (COP) in blood samples. However, an HAI titer is insufficient for capturing the whole entity of “flu immunity” [9], nor does it seem to be a good predictor of immunity in all age groups [10,11,12]. More recently, the analysis of responses to H3N2 viruses seem to be problematic in HAI assays [13,14,15]. In addition, widely used TIV/QIVs suffer from major obstacles, like a low vaccine efficacy (VE), especially in the elderly, and the need for annual vaccine adaptions due to the genetic instability of HA. As a result, current vaccines yield VEs below 70%, and can even approach zero if the vaccine does not match the circulating strain [16,17,18].

As early as 1944, an inactivated flu vaccine was developed by Thomas Francis and colleagues [19]. Remarkably, the basic principle of IAV vaccines produced in embryonated chicken eggs (ECEs) is still used nowadays, although significant problems arise from this vaccine production system, like the enormous demand for synchronized, pathogen-free chicken eggs, the time-consuming production cycle, mutations in the HA antigen due to egg adaption, or compatibility problems of some flu strains with ECE, to name a few. However, some improvements of TIV/QIV have been made lately. To increase VE in the elderly population, high-dose influenza vaccine formulations and specific adjuvants augment immunogenicity in this most vulnerable age group [20,21,22]. Cell culture-derived vaccines, like the recombinant influenza vaccine Flublok, generated in insect cells, and the inactivated mammalian cell-grown vaccine Flucelvax, entered the marked recently and decrease the demand for chicken eggs [23,24]. However, significant obstacles regarding the annual vaccine adaptions still exist with these technologies.

The need for seasonal adaptions also remains with LAIV. Such live-attenuated and temperature-sensitive IAV strains are produced by reverse genetics in chicken eggs, using six segments from the temperature-sensitive master donor strain and the HA/NA segments from the respective WHO vaccine recommendation. Administered as a nasal spray, these viruses can replicate to some extent in the colder upper respiratory tract, while they are not able to spread to the warmer lower respiratory tract [25,26]. Importantly, LAIVs induce not only classical HAI responses, but in contrast to TIV/QIVs, also local antibodies and cross-reactive T cell immunity specific for conserved internal influenza proteins. LAIVs thereby induce a broader immunity against divergent IAV [27,28]. However, while being a proof-of-principle for T cell-mediated heterologous immunity (Het-I), LAIVs do not present a vaccine option for all age groups, and they suffer from low VEs in some seasons [29,30,31].

## 3. Heterologous Immunity by T Cells

Due to the drawbacks of current flu vaccines, the research community has invested great effort into finding vaccine strategies that induce a universal or at least substantially broader immunity. T cell and antibody responses may both provide this broad immunity, but a combination of both might presumably be the most effective strategy. However, due to the topic of this review, we will focus on T cell-mediated Het-I. Others provide a more detailed picture about antibody-eliciting approaches [32].

As early as 1965, Jerome Schulman and Edwin Kilbourne observed that mice recovered from a previous H1N1 infection were partially protected against mortality, virus replication, and lung tissue damage following H2N2 infection [33]. Immunization with inactivated H1N1 was not able to induce Het-I, indicating that humoral responses are not sufficient for the protection. Later animal studies have proven the essential role of cross-reactive T cells for such infection-induced Het-I [34,35,36,37,38,39]. Almost forty years ago, McMichael and colleagues generated the first clinical data about Het-I by concluding from a human challenge study that cross-reactive, cytotoxic T cells can suppress nasal virus replication efficiently [40]. More recently, the 2009 H1N1 pandemic provided the unique chance to investigate the impact of pre-existing T cells on infections with a newly emerged pandemic IAV strain in a naturally exposed study population lacking protective antibody responses. One study detected IAV-specific T cell responses at baseline in 43% of the study population, and could prove that the presence of nucleoprotein (NP)-specific T cells correlated with a threefold decreased chance of acquiring a symptomatic, PCR-confirmed IAV infection [41]. Along this line, Sridhar et al. reported that pre-existing, cross-reactive T cells specific for polymerase basic protein 1 (PB1), matrix 1 protein (M1), and NP were inversely associated with illness severity in case of an infection with the novel H1N1 [42]. More specifically, the frequency of IAV-specific, cytotoxic (interferon-γ^+^, interleukin-2^–^), and lung-homing (CD45RA^+^CCR7^−^CD8^+^) T cells showed the strongest inverse correlation to symptom scores. CD4^+^ T cells were shown to correlate with disease protection as well [43]. In mice and humans, CD8^+^ T cell responses are predominantly focused on internal virus proteins like NP, polymerase (PA), PB1, polymerase basic 2 (PB2), and M1, while CD4^+^ T cell responses are more diverse, recognizing the surface proteins HA and NA as well [44,45,46,47,48,49,50,51,52,53,54,55]. Although the internal virus proteins are generally considered as more conserved than the surface proteins, viral escape through immune pressure can happen; however, it seems to be connected to a loss of viral fitness, as shown for NP [56,57].

## 4. Flu Immunity by Tissue-Resident Memory T Cells in Animal Models and Humans

When Schulman and Kilbourne conducted their studies in 1965, they could not know that they had observed T cell-mediated Het-I. This specific knowledge was just not available at that time. As immunology has made much progress in the recent decades, we can tell today that they not only did observe T cell immunity, but this immunity was probably also provided by tissue-resident memory T cells (TRMs).

Today we know that T cell responses are diverse in terms of functionality and spatial distribution. For CD8^+^ T cells, effector and effector-memory T cells (TEFFs and TEMs, respectively), central memory T cells (TCMs), and TRMs exist [58]. While the rather short-lived effector populations have access to peripheral tissues, including the lung, allowing them to directly fight invading pathogens by their cytotoxic functionalities, TCMs are longer-lived and predominantly circulate among lymph nodes to accelerate anamnestic responses in case of reinfection. In contrast to these circulating subsets, TRMs stably reside at the tissue of the primary infection [59,60].

Studies in mice have proven the essential role of lung CD8^+^ and CD4^+^ TRMs in providing Het-I against secondary IAV infections. Although localized T cell responses cannot prevent an initial infection like nAbs can do, they restrict virus replication, disease severity, and lung pathology [60,61,62,63]. However, CD8^+^ TRM responses in the lung tissue wane over time. Wu et al. found a substantial loss of TRM immunity within seven months after the primary infection [62]. Work from Takamura and colleagues suggests temporary TRM niches in the lung at the foci of tissue regeneration, because the disappearance of those repair-associated memory depots (the authors call them RAMDs) parallels the waning of TRM responses [60]. Moreover, lung CD8^+^ TRMs seem to be prone to apoptosis [64]. Interestingly, lung CD4^+^ TRMs occupy different niches than CD8^+^ TRMs in the airways and around B cell follicles, and seem to be more stably maintained [60,65,66].

TRM populations can differ phenotypically due to tissue-specific adaptions, but two commonly used markers are the C-type lectin CD69 and the integrin CD103. Both markers are expressed on murine CD8^+^ TRM cells in the respiratory tract [60,62,67]. Apart from this constitutive expression of adhesion molecules, the limited ability of TRM to recirculate is further caused by a lack of molecules that enable tissue egress and promote migration towards lymphatic tissues, such as S1PR1, CCR7, or CD62L. Accessory TRM markers like CD11a, CD49a, or PD-1 have been described as well [62,65,66,68]. However, the exact phenotype and the mechanisms that maintain specific TRM populations might differ depending on the inductive conditions. Even different immunodominant T cell clones against the same pathogen might show divergent transcriptomic profiles [67]. Lung CD4^+^ TRM populations and their phenotypes are substantially less well studied. Nevertheless, CD69 is a stringent marker for resident CD4^+^ T cells, while CD103 might indicate a regulatory phenotype rather than being a good marker for effector TRMs [61,69,70]. Apart from phenotypic occurrences, intravascular staining can be used to delineate TRMs from circulatory T cells [71].

Mouse models provide several advantages for immunological research, like the availability of tools and transgenic strains or economic husbandry. However, mouse models do not optimally represent human IAV disease, immunology, or lung anatomy. Thus, in a translational regard, other animal models might be more suitable to study TRM-mediated flu immunity. Particularly important for TRM research, methods to define and quantify localized T cell populations like parabiosis [59,60,61], intravascular staining [71], or specific phenotypic profiles were first established in mice, but can be principally transferred to less common laboratory animals (although parabiosis is technically difficult and ethically controversial). In the following, we will discuss the current knowledge about respiratory TRMs in other animal models and in humans (Table 1).

As early as 1933, ferrets were shown to be permissive to human influenza strains [91]. Nowadays, ferrets are a valuable model organism to study flu disease and novel vaccine candidates, due to their similarities in IAV pathogenesis and transmission compared to humans [92,93]. Immunologic studies in ferrets have been hampered so far by the lack of relevant reagents. However, efforts have been made to identify commercially available antibodies with cross-reactivity in the ferret model, leading to a basic selection of reagents [76,94]. A further expansion of this toolbox to analyze adaptive immune responses in ferrets is part of the strategic plan for a universal flu vaccine, communicated by the National Institute of Allergy and Infectious Diseases [95]. Several studies have shown that Het-I in ferrets induced by experimental infections, leading to reduced pulmonary virus replication and virus transmission to naïve ferrets [81,96,97,98,99,100,101,102,103]. TRMs were unfortunately not investigated in these studies. Also, a recent study demonstrated Het-I after H1N1 infection against a secondary H2N2 challenge [78]. Animals with pre-existing immunity experienced reduced virus replication, weight loss, and fever (a symptom not evident in mice, since they get hypothermic upon infection). The analysis of systemic T cells exposed cross-reactive responses biased towards the recognition of NP, non-structural protein 1 (NS1), and PA. Mucosal T cells were not analyzed after the priming infection. However, the authors established lung perfusion in ferrets to remove blood contaminations from the respiratory organ (although perfusion is not an optimal method to identify lung TRMs [71]) and isolated flu-specific T cells from the nasal turbinate and lung tissue after the secondary infection, which at least suggests the establishment of local T cells after IAV infections in ferrets. Intravascular staining or a rough phenotypic analysis, both feasible with available antibody clones against CD3, CD4, CD8, CD103, CD11a, and Ly6C [76], were not conducted.

H1N1, H1N2, and H3N2 strains are endemic in pig populations all over the world [104,105], and domestic pigs are a source of pandemic IAV strains, while also being economically of great importance. Therefore, this model organism is increasingly used in flu research, as outlined in a separate review [106]. Importantly, pigs show several anatomical and immunological similarities to humans [107]. Similar to the ferret model, the immunological toolbox is limited, but has grown in recent years for example by the identification of immunodominant T cell peptides and the development of respective MHC-multimers for NP, HA, NA, M1, and PB2-specific CD8^+^ T cells [80,108,109]. LAIVs are licensed for vaccination in pigs [110] and induce protection against matching and divergent IAVs [111,112,113]. T cell responses were not assessed in these studies, but others report an induction of T cells by LAIV vaccination [114,115,116] and by experimental infection with H1N2 [117]. Tungatt and colleagues used newly developed swine multimers to stain NP-specific CD8^+^ T cells in blood, lymph node, and bronchoalveolar lavage (BAL) samples of IAV-experienced pigs [80]. The latter ones showed the greatest T cell responses, with up to 13% multimer-positive cells. A separate study demonstrated that 90% of BAL T cells are protected from intravascular staining, suggesting that this population is mainly composed of TRM cells [81]. In contrast, T cells isolated from lung tissue contained only about 40% TRMs, indicating significant contamination by vascular T cells, as commonly described in mouse models [71]. Moreover, a rough phenotypic analysis of BAL TRM in pigs showed a predominant CD27^–^CCR7^–^ phenotype [82].

Non-human primates (NHPs) are the animal model with the highest degree of similarity to humans [118,119]. In particular, their adaptive immune systems are largely comparable and therefore NHPs present a valuable model for viral infections [120]. Likewise, human immunological reagents often show cross-reactivity to NHPs, resulting in a diverse toolbox. On the other hand, NHP models are expensive, and ethical aspects must be considered. This might be the reason why, despite great advantages, they are not used extensively to study (mucosal) flu immunity. Nevertheless, T cell-mediated Het-I has been reported in macaques [121,122]. The work of Pichyangkul and colleagues demonstrated a significant induction of local humoral and cellular responses following pulmonary exposure to the 2009 pandemic H1N1 virus [83]. NP-specific CD69^+^CD103^+^ lung TRMs (both CD4^+^ and CD8^+^) were highly prevalent in the lung, while only marginally found in blood. Another study described TRM phenotypes induced by *Mycobacterium tuberculosis* in macaques via intravascular staining and reported the expression of CD103 and CD69 on BAL und lung TRMs [84].

The analysis of localized T cell responses directly in human tissues is most meaningful regarding clinical applications. However, the investigation of human TRMs is difficult, since most immunological methods used in preclinical research are not applicable to humans. Thus, so far no direct investigation of TRM-mediated protection against flu infections has been conducted, but some insights could be generated in BAL samples, lung tissue biopsies [85,86,88,90], and human cadavers [74,87], as well as in the context of lung transplantation [123]. These studies have shown that CD4^+^ and CD8^+^ TRMs in the airways and lungs express CD69 and less stringently CD103. Other studies could further define accessory markers like chemokine receptors (CXCR3, CCR5, CCR6, CXCR6), adhesion molecules (CD49a, CD97), and checkpoint molecules (CTLA-4, PD-1) [86,88]. In a seminal study, Snyder et al. followed lung transplant recipients longitudinally for the maintenance of existing TRM phenotypes in the donor organ and the de novo generation of new TRM populations [89]. Donor TRM populations persisted for more than 15 months after lung transplantation, and expressed canonical TRM markers like CD69, CD103, CD49a, and PD-1. Two studies from Christopher Chiu’s lab investigated CD8^+^ and CD4^+^ TRM responses in experimental human respiratory syncytial virus (RSV) infection [85,90]. Immune responses were assessed longitudinally in BAL samples showing an accumulation of CD69^+^CD103^+^ TRM cells in the airways after convalescence. Moreover, the CD8^+^ TRM responses before the challenge correlated with reduced symptoms and viral replication. BAL CD4^+^ T cells were mainly CD69^+^, and about 20% showed additional expression of CD103. Thus, these studies reported protective effects of airway TRMs against human respiratory viruses for the first time. Similar experimental human challenge studies are essential to investigate TRM-mediated immunity against influenza.

## 5. Vaccine Strategies that Establish Pulmonary TRM Responses

In contrast to current IAV immunization strategies, which primarily induce humoral responses, numerous preclinical vaccine candidates exploit T cell immunity to induce protection against a broad spectrum of IAV strains. Considering the important contribution of localized T cell responses to Het-I, several strategies aim at the induction of TRM responses in the respiratory tract. A main prerequisite for respiratory TRMs is a local delivery of antigens [60,62,63] or specific adjuvants that bypass the need for local antigens [124]. Nevertheless, one of these vaccine components must be administered into the airways. Moreover, an induction of lung CD8^+^ TRMs relies on antigen cross-presentation by dendritic cells (DCs), mainly by migratory CD103^+^ type I conventional DCs [125]. This antigen cross-presentation is much more efficient after genetic vaccination, leading to endogenous antigen production in the host, compared to protein- or peptide-based strategies that need further enhancement by adjuvants or other mechanisms.

Several genetic vaccine platforms have been established for intranasal delivery, such as DNA formulated with polyethylenimine [126,127], adenoviral vectors [75,128,129,130,131,132], recombinant Sendai virus [133,134], modified vaccinia Ankara virus (MVA) [135], or murine cytomegalovirus vectors [136]. By the vector-driven expression of conserved internal flu proteins, all these approaches (and many more not mentioned here) are able to induce Het-I. However, the extent of lung TRM establishment might differ among these platforms. Even vectors based on different subtypes of the same virus family evoke divergent immune profiles [137]. Thus, an induction of lung TRMs per se might not be problematic with genetic vaccines, but the amplitude and the long-term maintenance are parameters to be improved by refined strategies. First, an increased number of initial TRMs could lead to longer maintenance of the protective levels of T cell immunity. The co-delivery of inflammatory factors in genetic vaccinations has been used extensively to modulate systemic immune responses [138,139,140,141,142,143,144,145]. We could show that the mucosal co-delivery of interleukin (IL)-1β can increase the induction of lung CD4^+^ and CD8^+^ TRMs significantly [73], thus illustrating that genetic adjuvants might be an important tool to boost TRM responses. Second, repetitive intranasal immunizations over time can maintain and refresh the existing TRM pool [146]. Third, optimized heterologous prime-boost regimens or even simultaneous immunizations at different body sites might lead to synergistic effects between local and systemic immune responses to ensure long-term immunity [147].

When it comes to protein- and peptide-based vaccines, additional tricks must be exploited to enhance the induction of T cell immunity in the respiratory tract, especially regarding CD8^+^ TRMs. Some of these tricks rely on the use of specific adjuvants or antigen-targeting to cell types of interest. Wakim and colleagues used an antibody-targeted intranasal vaccination to specifically address a model antigen to CD103^+^ DCs. As a result, antigen-targeting to Clec12a or Dec205 could increase induction of CD103^+^ CD8^+^ TRM cells in the lung, leading to improved protection against a subsequent IAV challenge [148]. Another study from the same lab describes the use of zymosan as a mucosal adjuvant, allowing an establishment of CD103^+^CD69^+^ CD8^+^ TRMs independent of local antigen application to the lungs [124]. However, both studies used a model where in vitro activated, T cell receptor-transgenic T cells were administrated before immunization. The physiological priming process of naïve CD8^+^ T cells was not assessed in these models. Therefore, it would be interesting to see whether these strategies work in a less artificial vaccination setting as well. Another study used the conserved matrix 2 protein ectodomain fused to a protein-adjuvant called CTA1-DD (cholera toxin A1 derivative and *S. aureus* fragment D dimer) as an intranasal vaccine [69]. The vaccine induced CD103^low^ CD69^+^ CD4^+^ TRM responses that were crucial for flu immunity, and the protective effects partially relied on IL-17A production. These data illustrate the important role that CD4^+^ TRMs can play in Het-I and the feasibility to induce protective levels of CD4^+^ TRMs by protein vaccines.

## 6. Clinical Studies of T Cell-Inducing Flu Vaccines

While numerous vaccine strategies that evoke cross-reactive T cell responses have been developed in animal models, a fairly low number also progressed towards human clinical trials. Even fewer of these studies have exploited local or mucosal administration strategies. A comprehensive review about clinical trials with universal flu vaccine candidates is given elsewhere [149]. Here, we will predominantly focus on vaccines intended or expected to induce T cell responses and for which clinical data have already been published.

A few trials evaluated epitope-based peptide vaccines, but so far none of them have induced efficient protective immunity. Flu-v consists of conserved peptides derived from M1, M2, and NP formulated with Montanide ISA-51 as an adjuvant. The vaccine induces cellular immunity in humans, and could reduce symptoms and viral shedding in a small human challenge study [150,151]. A recently published phase IIb study showed only a limited capacity of the vaccine to protect against mild to moderate influenza disease in experimental infections. One but not two vaccine doses reduced disease burden, but an impact on virus shedding could not be found in any of the vaccinated groups [152]. BiondVax’s Multimeric-001 peptide vaccine contains nine conserved B cell, CD4^+^, and CD8^+^ T cell epitopes from HA, NP, and M1 of IAV and IBV [153]. While it has been proven safe and immunogenic, recent data from a phase III trial (NCT03450915) with more than 12,000 participants did not demonstrate significant VE against flu infections [154]. Another peptide vaccine is FP-01.1, which combines peptides from NP, M1, PB1, and PB2. Two doses of the vaccine, given four weeks apart, resulted in a responder rate of 83% in the high-dose group. CD4^+^ and CD8^+^ T cell responses against the conserved vaccine immunogens were induced and peaked at day 7 post-immunization. By now, no data about longevity of the immune responses or VE were published, but an experimental challenge study was conducted (NCT02071329).

If the induction of strong CD8^+^ T cell responses is desired, genetic immunizations with DNA, RNA, or viral vectors that lead to endogenous antigen production in the vaccinees have an intrinsic advantage over protein- and peptide-based vaccines. One of the most characterized vector-based universal flu vaccine approaches is MVA–M1/NP. In total, nine clinical trials were initiated with this vaccine candidate, which encodes full-length NP and M1 derived from IAV. MVA–M1/NP was shown to induce CD4^+^ and CD8^+^ T cell responses specific for the conserved vaccine epitopes. Analyses of the vaccine immunogenicity in different age groups presented a decreasing induction of T cell responses in older individuals. While vaccine-induced T cell responses remained statistically significant over the baseline for 52 weeks in 50–59-year-old participants, the responses in >70-year-old participants were only significant over the baseline for three weeks after vaccination [155]. In a human challenge study, the vaccine showed a slightly decreased infection rate in the vaccine group (2 out of 11) compared to the placebo group (5 out of 11), but an interpretation of the vaccine effect was hampered by the unexpectedly low number of infections in the placebo group [156]. Of note, the flu infections in the placebo group induced stronger T cell responses than vaccination with MVA–M1/NP, suggesting that vaccine-induced immunity is weaker compared to naturally acquired immunity. VE is currently being evaluated in larger trials, both as a standalone vaccine (NCT03883113) and as adjunct to a licensed QIV (NCT03880474). ChAdOx1–NP^+^M1 is based on a replication-deficient chimpanzee adenovirus. Similar to the MVA–M1/NP, it encodes full-length NP and M1 proteins. In a phase I study, the vaccine was shown to induce T cells against the vaccine antigens, peaking 14 days after immunization. A boost with the aforementioned MVA–M1/NP 7 or 14 weeks after ChAdOx1–NP^+^M1 could further increase T cell responses [157].

While all the above mentioned vaccine trials exploited systemic immunization routes, mucosal vaccine administration is key to engage local immune responses, especially TRMs in the respiratory tract. Altimmune’s NasoVax is an adenovirus serotype 5-based vaccine (Ad5) encoding full-length HA from an H1N1 strain. No trial data have been published yet, but results presented at a conference suggest superior immunogenicity with a high dose of NasoVAX compared to a licensed QIV [158]. Besides a 100% seroprotection rate, T cell responses were six-fold higher compared to the QIV. It must be investigated to which extent those responses provide Het-I, but cross-reactive T cell responses against conserved epitopes of HA are described [159,160].

In addition, two orally given vaccines have been in human trials. One trial was assessing replication-competent adenovirus serotype 4 (Ad4) encoding HA from an H5N1 strain [161]. The “vaccine take”, defined as the percentage of vaccinees being PCR-positive and/or seropositive for Ad4 after vaccination, was strongly dose-dependent but reached 96% after three high-dose immunizations. A total of 70% of the participants in these high-dose groups mounted antigen-specific T cell responses, but these consisted predominantly of CD4^+^ T cells. While HA seroconversion was achieved in only a minority after Ad4–HA immunization (4–19%), a parenteral boost vaccination with inactivated H5N1 led to increased seroconversion rates in a dose-dependent manner like the previous Ad4–HA immunizations. Similarly, an Ad5-vectored vaccine encoding HA from H5N1 from VaxArt induced T cell responses in 75% of the participants, but failed to induce nAbs [162]. Unfortunately, the specific contribution of CD4^+^ and CD8^+^ T cells were not deciphered in these analyses. It is important to note here that the low seroconversion rates reported in these studies are not due to a low immunogenicity of the vaccine platforms; instead, this is rather attributed to a generally low immunogenicity of some avian HA variants [163].

Another vaccine from VaxArt, VXA-A1.1, relies on a non-replicating Ad5 encoding HA and an immunostimulatory dsRNA as a TLR3 agonist. VXA-A1.1 is given as a tablet and targets epithelial cells in the small bowel [164]. Due to the tablet formulation and its stability at room temperature, this vaccine enables distribution independent of healthcare professionals, for example by mail delivery. Moreover, it seems that the oral administration evades pre-existing anti-vector immunity to some extent. The respond rate after one dose of the vaccine was 92% with regard of humoral responses (four-fold increase in HAI) [164]. In a recent phase II study, the VE against a vaccine homologous strain was evaluated by experimental infections. Compared to a study group that received a licensed QIV, VXA-A1.1 generated a similar protective immunity against infection [165]. Since serum HAI titers were about nine-fold higher in the QIV group compared to VXA-A1.1, additional immune parameters must be responsible for the observed VE. Immunoglobulin A (IgA)- and immunoglobulin G-secreting cells, as well as polyfunctional T cells, have been correlated with protective immunity. Mucosal immunity in the respiratory tract was not directly assessed in this study, although preclinical studies report an induction of mucosal IgA in the respiratory tract [166]. Whether orally administered vaccine platforms are able to establish respiratory TRMs has not been reported so far, but the “gut–lung axis” allows some tissue-resident immune populations to traffic between both organs [167].

In conclusion, vaccine strategies aiming at the induction of cross-reactive T cell immunity have been evaluated in clinical trials, but no breakthroughs with clear and effective Het-I were reported so far. Some vaccines induce T cells but lack efficacy; others show a relatively rapid decline of T cell immunity. While a few mucosal vaccines have entered clinical trials, none of the studies really assessed local immune responses directly. However, the analysis of vaccine-induced TRMs is essential to define new mucosal correlates of protection that are not accessible in peripheral blood mononuclear cells (PBMCs).

## 7. Challenges in Establishing TRMs as Protective Correlate

Preclinical studies have illustrated the indispensable role of localized T cell responses in the protection against IAV. Concomitantly, many different vaccination strategies have been developed in these animal models to establish TRMs and eventually led to broad and effective Het-I. However, it is still a long way to establish T cell immunity as a protective correlate in humans. In the following, we will discuss essential steps to define novel COPs and give an outlook about a potential implementation of T cell immunity in current vaccination guidelines.

Early-phase clinical trials are not only important to show the safety of novel vaccines, but are also key to estimate their efficacy and to prioritize on the most promising candidates for later phases. To this end, either a direct correlate of protection or a surrogate marker must be assessed to interpolate protective efficacy from vaccine immunogenicity. Classically, the HAI is such a correlate with a clearly defined protective threshold, although there are debates about the general validity of a hemagglutination inhibition titer of 1:40 for protective immunity [12,168]. Such specific thresholds of protective immunity parameters (directly assessed COPs or surrogate parameters) are urgently needed for T cell-mediated flu immunity.

The first obvious question is this: where should T cell responses be sampled? While PBMCs might be an adequate biological sample to estimate systemic T cell immunity, mucosal vaccines hold the greatest promise to evoke local immune responses. Thus, sampling at the mucosal site of interest is key to evaluate the actual purpose of these vaccines. However, what is the actual mucosal site we would like to address? In animal models, lung TRMs are the most studied mucosal T cell COP [62,63]. However, it is unlikely that human vaccines will exploit administration routes directly aiming at the lower respiratory tract, due to the invasive nature of such procedures and potential side effects. Instead, it is more likely that mucosal next-generation vaccines will target tissues of the upper respiratory tract, like the nasal mucosa. In mice, CD8^+^ TRMs in the nasal epithelia were shown to protect against severe disease by blocking pulmonary spread of the IAV infection [169]. Sampling of nasal tissue in clinical trials would be much less elaborate compared to sampling of the lung tissue. However, human challenge studies with RSV as well as studies from the Farber lab illustrate the feasibility of assessing CD4^+^ and CD8^+^ TRM responses in the lower respiratory tract by bronchial biopsies and BALs in humans [85,89,90]. An interesting alternative induction and sampling site for mucosal immunity are nasopharynx-associated lymphoid tissues, which include the adenoids and tonsils. In particular, studies from Rebecca Cox showed that immunization with LAIV in children rapidly evokes B cells, CD4^+^ T cells, and CD8^+^ T cells in the tonsils [170,171]. While it seems clear that tonsillar T cells are induced upon intranasal LAIV immunization, and that these responses correlate with serum HAI, their contribution to or significance in mucosal immunity is not clear yet. Neither their direct protective effect (used as direct COP), nor any correlation with tissue TRM responses (used as surrogate marker) have been described so far. Interestingly, tonsillar immunization with genetic vaccines in NHPs induce T cell responses in BAL samples, which are considered as a stringent TRM population [172]. Thus, there are several mucosal sites where immune responses can be induced and assessed. Future studies must investigate the relevance of each of these compartmentalized responses in respect to protection.

Preclinical animal models are essential to investigate the basic immunological principles of anti-flu immunity and have helped us in the past to develop concepts like T cell cross-reactivity, TRM responses, and universal flu vaccine approaches. However, the critical step is to translate these concepts into real-life human vaccines. A combination of controlled human challenge studies that employ in-depth immunological analyses and large efficacy trials seem to be required to establish novel COPs. This was also outlined at the “Immunological Assays and Correlates of Protection for Next-Generation Influenza Vaccines” meeting in 2019 [173]. Once specific COPs are identified, it is important to define protective thresholds of the respective responses (absolute COPs). For systemic T cell responses, Forrest et al. found that >100 spot-forming cells per 10^6^ PBMCs in interferon-γ ELISPOT analyses correlated with protection against symptomatic flu infections in young children vaccinated with LAIV [174]. Although this value is discussed within the field, this type of protective threshold is needed to estimate VE in clinical trials from immunogenicity data. Likewise, the sample collection at different (mucosal) body sites and the actual immunological assays must be standardized to allow direct comparisons of clinical trials among study sites and labs [173]. This also helps to prioritize the most promising vaccine candidates already after early clinical phases.

## 8. Consideration of Cellular Immunity in Vaccination Practices

Once effective flu vaccines that rely on T cell responses are market-ready, it must be discussed how those are integrated in vaccination practices and recommendations. Elderly people aged over 65 years, people with underlying diseases (diabetes, chronic obstructive pulmonary disease, asthma, heart and kidney diseases), and pregnant women are the most vulnerable groups. Since sterilizing immunity is the most efficient way to protect them against flu complications, the induction of nAbs must remain the most important COP in these risk groups. Moreover, the induction of nAbs in pregnant women is important for a maternal antibody transfer to the offspring—an important risk group that is unlikely to receive genetic or mucosal flu vaccines within the first months of life.

All parts of the community would benefit from the presence of cross-reactive T cell immunity against flu. In risk groups, T cell immunity would present a safeguard in case of vaccine mismatches and emerging pandemic strains. Young and healthy individuals do not necessarily require sterile protection against influenza, since potential infections in these groups are less likely to result in severe disease. Nevertheless, cross-reactive T cell responses can further decrease the rate of flu-related complications and might also to some extent provide population-wide protection against emerging flu strains with higher pathogenicity. At the same time, the non-sterile nature of T cell immunity would still allow mild IAV infections, which can naturally boost systemic and mucosal T cell responses [175]. Thus, regular boosting of T cell immunity by T cell-inducing vaccines and natural infections might be a way to maintain long-term mucosal Het-I in humans. Table 2 summarizes our recommended vaccination practices for different target groups.

So far, the effect of such broad and pronounced T cell immunity in large parts of the community on virus evolution is unknown. As pointed out by others, one result could be a general decrease of flu infections, while decreasing virus replication per person is also slowing down virus evolution. On the other hand, immune pressure on conserved virus proteins exceeding natural immunity could select for escape mutants [176]. However, viral escape might be limited due to functional constraints and loss of viral fitness [57].

Regarding the vaccine platforms, a full switch to genetic and favorably mucosal vaccines in all target groups should take place if the safety profile of the respective vaccine allows it. In those vaccines, the antigen components can be combined and updated as needed. All vaccine formulations should include conserved flu proteins like NP, M1, or polymerase proteins in order to induce cross-reactive T cell responses. In the mentioned risk groups, HA-encoding components can be easily added (and adapted annually) in order to evoke nAbs, preferentially in the respiratory tract. This strategy combines the advantages of genetic vaccines regarding immunogenicity, manufacturing, and adaptability, while it considers the vulnerability of specific target groups and the benefits of infection-permissive immunity in healthy individuals at the same time.

## 9. Concluding Remarks

Recent influenza vaccines are not appropriate to protect the community against seasonal and pandemic flu strains. The time has come to implement modern immunology and vaccine technology in human flu vaccines. In the context of the latest Ebola and SARS-CoV-2 outbreaks, several efficient genetic vaccines have been approved. Thus, the aim should be to employ these technologies for routine flu shots also in order to enable new T cell-based COPs. Many years of preclinical research prove the protective potential of cross-reactive T cell immunity and more recently of respiratory TRMs. Substantial knowledge could be gathered to understand and induce TRM responses in animal models. It is now critical to illuminate remaining knowledge gaps and their translation into clinical approaches. Current preclinical data indicate that local inflammation in the respective mucosa is a minimal prerequisite for the establishment of local T cells. Local expression of antigens seems to be additionally required for the induction of lung TRMs. Therefore, local vaccination techniques currently seem inevitable to evoke TRM responses. However, the respiratory tract is an immunologically fragile environment, and the unintentional induction of autoimmunity or allergies must be avoided. This becomes even more important if mucosal vaccines are used in individuals with pre-existing respiratory diseases. For the long-term maintenance of immunity, it is essential to develop vaccine strategies that either induce long-lived TRM populations or refresh short-lived TRMs regularly. Eventually, the consideration of novel vaccine technologies and cross-reactive T cell responses holds the promise of decreasing flu mortality in seasonal and pandemic outbreaks significantly.

## Figures and Tables

**Table 1:** Current knowledge about respiratory tissue-resident memory T cells (TRMs) in different species.

	Respiratory TRMs Reported	Methods Used to Define TRMs	Phenotype (Accessory Markers)	Induced by	Contribution to Het-I	Refs
Mouse	Yes, CD4^+^ and CD8^+^	Intravascular staining, parabiosis, phenotyping, transcriptomics	CD69^+^CD103^+/−^ (CD11a, CD49a, PD-1, CD127, CXCR6)	Infection, vaccination	Yes, both CD4^+^ and CD8^+^ TRMs provide Het-I	[59,60,61,62,72,73,74,75]
Ferret	Yes, CD4^+^ and CD8^+^	Lung perfusion to exclude vascular T cells	n.d., but tools available	Infection	Correlation	[76,77,78]
Domestic pig	Yes, CD4^+^ and CD8^+^	Intravascular staining	(CD27^–^CCR7^–^)	Infection, vaccination	Correlation	[79,80,81,82]
NHPs	Yes, CD4^+^ and CD8^+^	Intravascular staining, phenotyping	CD69^+^CD103^+/−^	Infection	n.d.	[83,84]
Human	Yes, CD4^+^ and CD8^+^	Phenotyping, transplantation, transcriptomics	CD69^+^CD103^+/−^ (CD49a, CXCR3, CCR5, CCR6, CXCR6, CD101, CD97, CTLA-4, PD-1)	Pre-existing, infection	n.d. (correlation of airway CD8^+^ TRMs in RSV clearance)	[74,85,86,87,88,89,90]

n.d.: not determined; NHPs: Non-humane primates; RSV: respiratory syncytial virus.

**Table 2:** Implementation of T cell immunity in vaccination practices.

Target Group	Sterilizing Immunity Desired ?	T Cell Immunity Desired ?
Young children 0–2 years	Yes, preferentially by maternal antibodies in the first months of life	Yes, but approval of genetic vaccines might be difficult in this age group
Healthy individuals 2–65 years	No	Yes, by genetic vaccinations and natural infections
Elderly > 65 years	Yes	Yes, by genetic vaccinations
Adults with chronic health conditions	Yes	Yes, by genetic vaccinations
Pregnant women	Yes, to protect during pregnancy and for the transfer of maternal antibodies	Yes, preferentially induced before conception
